# Development of a conceptual framework for a group-based format of the Lifestyle-integrated Functional Exercise (gLiFE) programme and its initial feasibility testing

**DOI:** 10.1186/s40814-019-0539-x

**Published:** 2020-01-22

**Authors:** Franziska Kramer, Sarah Labudek, Carl-Philipp Jansen, Corinna Nerz, Lena Fleig, Lindy Clemson, Clemens Becker, Michael Schwenk

**Affiliations:** 1 Network Aging Research (NAR), Heidelberg University, Heidelberg, Germany; 2 Department of Clinical Gerontology and Geriatric Rehabilitation, Robert Bosch Hospital, Stuttgart, Germany; 3 Faculty of Natural Sciences, Department of Psychology, Health Psychology, Medical School Berlin, Berlin, Germany; 4 Faculty of Health Sciences, University of Sydney, Sydney, Australia

**Keywords:** Older adults, Fall prevention, Functional balance and strength training, Health behaviour change, Habit formation, LiFE, Lifestyle-integrated exercise

## Abstract

**Background:**

The Lifestyle-integrated Functional Exercise (LiFE) programme is a fall prevention programme originally taught in a resource-intensive one-to-one format with limited feasibility for large-scale implementation. The aim of this paper is to present the conceptual framework and initial feasibility evaluation of a group-based LiFE (gLiFE) format developed for large-scale implementation.

**Methods:**

The conceptual gLiFE framework (part I) is based on three pillars, *LiFE Activities and Principles*, *Theory of Behaviour Change and Behaviour Change Techniques*, and *Instruction*. The feasibility of gLiFE was tested (part II) within a multimodal approach including quantitative questionnaires measuring safety, acceptability (1 = best to 7 = insufficient), and adherence to the LiFE activities (range = 0–14) as well as a focus group interview. Exploratory self-reported measures on behaviour change including self-determined motivation (range = 1–5), intention, planning, action control, and habit strength (range = 1–6) were assessed pre and post intervention. Data analyses were performed using descriptive statistics and qualitative content analysis.

**Results:**

The development process resulted in a manualised gLiFE concept containing standardised information on gLiFE’s content and structure. Feasibility testing: Six older adults (median = 72.8 years, 5 female) completed the feasibility study and rated safety (median = 7.0, IQR = 0.3) and acceptability as high (median = 1, IQR = 1). Participants implemented 9.5 LiFE activities (IQR = 4.0) into their daily routines. No adverse events occurred during the study. In the focus group, the group format and LiFE activities were perceived as positive and important for maintaining strength and balance capacity. Self-determined motivation intention, planning, and habit strength were rated higher post intervention.

**Conclusion:**

The developed conceptual gLiFE framework represents the basis for a gLiFE format with potential for standardised large-scale implementation. Proof-of-concept could be demonstrated in a group of community-dwelling older adults at risk of falling. The public health potential of gLiFE in terms of (cost-)effectiveness is currently being evaluated in a large trial.

**Trial registration:**

ClinicalTrials.gov NCT03412123. Registered on January 26, 2018

## Introduction

Since falls display a major health risk factor in our ageing society [1–3], there is a strong need for increasing accessibility to effective fall prevention programmes. Across different settings, multifactorial training, such as the combination of balance and strength exercises have shown to be most effective in reducing fall rates in older adults [4–8]. However, the “traditional” delivery of balance and strength exercises through structured training often entails low long-term adherence of participants [9–11]. Lifestyle-integrated training was developed as an alternative approach in order to increase long-term adherence through embedding functional exercises into daily life, that is, daily routines are enriched with small low-intensity bouts of activity with the aim to create new activity habits [12–14]. Lifestyle-integrated training has already shown positive effects on fall-related outcomes [12, 15]. For example, the Lifestyle-integrated Functional Exercise (LiFE) fall prevention programme by Clemson et al. [16] recorded greater adherence rates compared to a traditional, structured training. LiFE resulted in a greater increase in motor performance, physical activity and a greater decrease in fall rate compared to the comparator groups. Despite its high potential, LiFE’s large-scale implementability is hampered by its resource-intensive one-to-one delivery format within seven home visits [17–19]. A promising solution could be delivering LiFE in a group format (gLiFE).

Three pilot studies on developing a group-based LiFE have already been conducted [20–23]. These group-based concepts were not specifically designed for large-scale implementation. For instance, Gibbs et al. [21, 22] developed a LiFE concept combining four group sessions and one individual session. The individual session aimed tailoring the LiFE activities to participants’ individual home environments. While such tailoring is justifiable from a scientific point of view, the additional resources needed conflict with the aim of cost-efficient large-scale implementation. The question is whether tailoring LiFE to a home environment can also be achieved in group sessions, for instance by applying specific teaching methods such as visualisation or group discussions about the individual home environment.

Another study [20] used three trainers to implement group-based LiFE in a sample of 13 young seniors (59–61 years). The high trainer-participant-ratio ensured optimal teaching of the LiFE concept (including one-to-one consultations during group sessions) and a high level of safety during exercising. The high resources needed for this group-based concept may hamper large-scale implementation. The question is whether specific teaching methods and optimal organisation forms may allow for a lower trainer-participant-ratio, without loss of teaching quality and safety. In summary, even though the current group approaches provide a valuable scientific contribution, a group LiFE concept for large-scale implementation needs to be developed and evaluated.

Important features for a gLiFE concept designed for resource-saving public health implementation are an optimised trainer-participant-ratio, implementability into different settings (e.g., community college, community centre), and portable low-cost material allowing quick and easy implementation by group trainers. Further, a standardised trainer’s manual could provide comprehensive pathways for teaching both the LiFE strength and balance activities and behavioural change. Such manual is fundamental for standardised large-scale implementation.

Apart from the lack in focus on large-scale implementability, current group-based LiFE formats [20, 24] show room for improving the delivery of behaviour change content. The fundamental aspect of long-term maintenance of the LiFE activities could be reinforced by emphasising on habit formation. Refinements should be made from a large-scale implementation perspective and brake down complex behaviour change theories to comprehensive units. This could enable cost-efficient teaching of programme content by providing for the trainers and therapists (e.g., physical or occupational therapists) a stronger understanding of the psychological underpinnings of the programme.

The aim of this paper is twofold: to present a newly developed gLiFE concept focused on large-scale implementation and building on a sound theoretical framework with a stronger focus on behaviour change (part I) and to present results of an initial feasibility testing of this new gLiFE concept (part II).

### Part I: conceptual framework of gLiFE

The conceptual gLiFE framework was developed building on existing LiFE concepts [20, 24] and theories and methods on group learning. Specific behaviour change theories were used to refine the theoretical framework in order to support long-term maintenance of LiFE. gLiFE was developed (part I) and initially tested in a feasibility study described in this paper (part II). The cost-effectiveness evaluation of gLiFE within a non-inferiority trial (grant no. 01GL1705A-D) comparing gLiFE to LiFE is currently being carried out and not described in this paper. The study protocol is described elsewhere [17].

### gLiFE development process

The development process was based on the UK Medical Research Council (MRC) guidelines [25] which propose four steps (development, feasibility and piloting, evaluation, implementation) for the design of complex interventions. An interdisciplinary team of experts in exercise science, health and social psychology, occupational therapy, geriatric medicine, physiotherapy, health economy, and gerontology took part in the development of the gLiFE concept. In addition, 11 users aged 67 to 90 were involved to test and evaluate possible forms of gLiFE during the development process.

Based on previous LiFE studies [18, 19, 24], the number of seven group sessions and sequence of gLiFE activities was determined. In order to compensate for a lower trainer-participant-ratio, theories [26, 27] and methods [28–32] on group learning informed the design of the framework conditions including group size [31], organisational setting [28] and structure [29–31]. The way of instructing gLiFE was informed by the Social Learning Theory [27] which proposes role models and reinforcement as core elements of the group learning setting. Through group activities and discussions, gLiFE fosters group cohesion [26] in order to keep participants engaged and motivate them to practice LiFE.

The development process resulted in a manualised gLiFE concept containing relevant information on content and structure of each gLiFE session. Table 1 provides an overview of the modifications undertaken in gLiFE compared to the individually delivered LiFE.

**Table 1:** Similarities and differences between LiFE and the newly developed gLiFE format

	LiFE	gLiFE
Aim	Improve balance and lower limb strength, increase physical activity, decrease risk of falling; long-term sustainability of the LiFE activities through habit formation and self-empowerment	
Idea	Create new movement habits through linking LiFE activities to specific daily situations	
Structure	Up to seven home visits of 1 hour; explain the LiFE principles during the first home visit, introduce the LiFE activities flexibly (1–2 balance/strength activities per session)	Seven sessions of 2 hours; introduction of LiFE activities in a predetermined order
Content	LiFE principles, balance and strength activities, adapt activities to own training progress (upgrading)	
	Planning	Planning (implementation intentions), theory-based behaviour change units, group discussion
Teaching	Foster autonomy in choosing daily situations for implementing the LiFE activities; tailor and adapt the LiFE activities throughout the intervention phase, visualisation	
Instruction	Flexible procedure	Detailed curriculum (gLiFE concept), trainers follow teaching methods (e.g., repetition and variation) and BCTs^a^, different organisational settings (mostly circle of chairs)
Materials	LiFE assessment tool (assessment of level of difficulty in movement execution), LiFE participant’s manual	
	Activity counter (recording the number of performed activities), activity planner (detailed planning on when, how, and where the activities can be implemented), daily routine chart (identify suitable opportunities for implementing LiFE activities into daily routines)	Workbook (including activity counter and activity planner), flipchart, posters, cardboard boxes, and towels
Setting	Participant’s homes	Public room
Trainer-participant-ratio	1:1	1:6 (two trainers in a group of up to twelve participants)

^a^Behaviour Change Techniques (BCTs) are the smallest identifiable parts of behaviour change interventions, mapped by Michie et al. (2011)

### Conceptual gLiFE framework

**Fig. 1:**
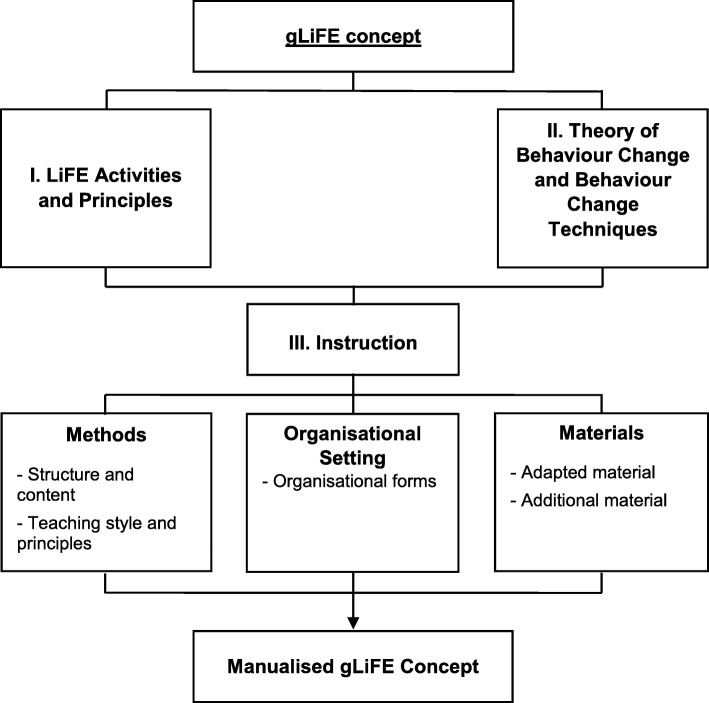
Conceptual gLiFE framework. The first pillar *LiFE Activities and Principles* is based on the original LiFE activities and principles which are “reducing your base of support”, “shifting weight and moving to the limits of stability”, “stepping over objects” [33] for balance and “increase the number of times that you use a muscle”, “move slowly – this can make the muscles work harder”, “use fewer muscles to move the same weight”, and “increase the amount of weight you have to lift or move” [33] for strength. The second pillar *Theory of Behaviour Change and Behaviour Change Techniques* is novel to gLiFE and provides a theoretical underpinning using the Health Action Process Approach, habit formation theory, and Self-Determination Theory as well as a conceptualisation and description of gLiFE’s components with the help of the BCTs. The third pillar *Instruction* consists of methods, organisational setting and materials and describes the way of delivering gLiFE. In the manualised gLiFE concept, a detailed curriculum is provided in order to teach gLiFE in a standardised manner

The conceptual gLiFE framework is based on two main pillars, *LiFE Activities and Principles* and *Theory of Behaviour Change and Behaviour Change Techniques* (Fig. 1). The third pillar, *Instruction*, predefines how the contents of gLiFE are delivered. The subcategories *Methods*, *Organisational Setting*, and *Materials* contain more detailed information on how to carry out gLiFE.

#### Pillar I: LiFE activities and principles

The content of the LiFE programme, the LiFE activities and principles from Clemson et al. [16, 33] were used as a foundation for gLiFE. LiFE contains 14 activities addressing static and dynamic balance, lower limb strength, and overall physical activity. These activities are effective for the target group of fall-prone older adults but at the same time performable during daily activities. Teaching the LiFE principles (Fig. 1) alongside the LiFE activities enables participants to integrate the activities into their daily routines and manage their training independently and sustainably [24].

**Fig. 2:**
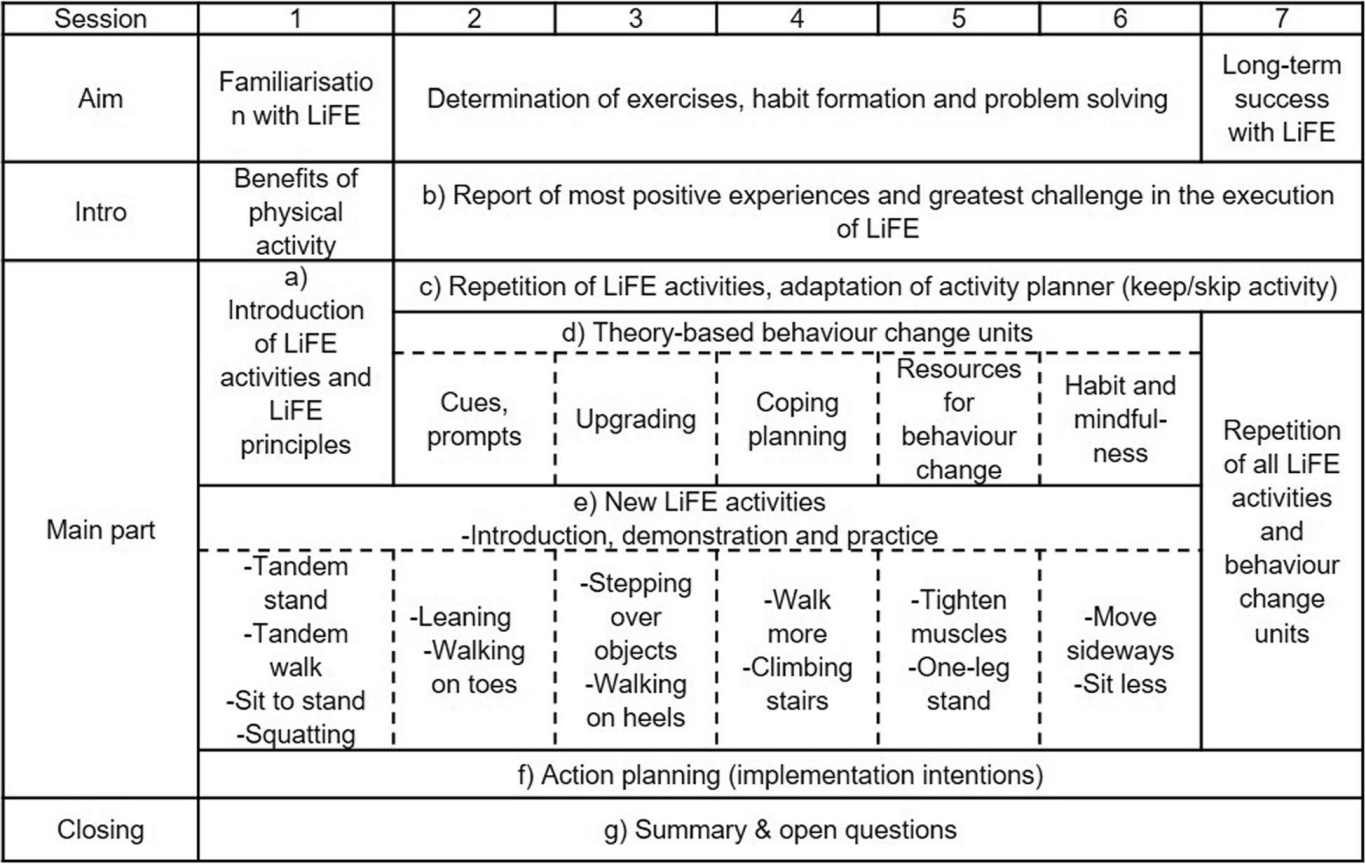
(**a**) to (**g**) refer to the chronological introduction of categories in the text. The LiFE Activities and Principles (pillar I in the conceptual gLiFE framework) are addressed in section (**a**), (**b**), and (**e**). Theory of Behaviour Change (pillar II) is addressed in section (**d**) and Behaviour Change Techniques (pillar II) infuse all gLiFE sessions. gLiFE contents are matched with the BCTs in Table 5 in Appendix 1

In LiFE, there is no predefined order along which the LiFE activities should be introduced. Experiences from the user involvement showed that teaching the LiFE activities to a group requires a different approach. Therefore, a standardised order of introducing LiFE activities over the course of the seven sessions was developed (Fig. 2). The order of LiFE activities was determined based on user preferences evaluated in a previous study [19]. In gLiFE, the most popular LiFE activities which are easy to integrate (e.g., sit to stand) are introduced during the first group sessions. More complex activities (e.g., stepping sideways) and more challenging activities (e.g., one-leg stand) are introduced later. Gradually increasing the complexity of content taught over the course of the group sessions aims to prevent overtaxing participants and ensures positive learning experiences.

#### Pillar II: theory of behaviour change and behaviour change techniques

LiFE goes beyond traditional fall prevention programmes; it aims for the establishment of new movement habits through integrating exercises into daily routines [24]. The theoretical underpinning of gLiFE was formed using the existing conceptual model of LiFE [24, 33], habit formation theory [34, 35], and the pilot study of Fleig et al. [20] which used the Health Action Process Approach (HAPA) [36, 37]. Additionally, we used the Self-Determination Theory [38].

Habit formation theory describes the habit formation process within three subsequent stages: intention formation, action initiation and habit formation [39]. After deciding to act, a person needs to apply self-regulatory strategies in order to act out the behaviour. After various repetitions of the behaviour in the same context, the association between the context and the behaviour strengthens until the execution becomes automatic (habitual). gLiFE makes use of this mechanism so that participants perform the LiFE activities habitually in the long run.

The HAPA served to enrich habit formation theory because of its emphasis on the motivational and volitional factors during behaviour change. These factors are particularly relevant during the early stage of behaviour change and could provide additional support for beginners. For example, the planning procedure was specified by using implementation intentions [40]. Instead of stating how, when and where to perform the LiFE activity, participants explicitly formulate a whole sentence in which the situational cue is followed by the LiFE activity (e.g., "If I brush my teeth, then I do the tandem stand"). This novelty may promote habit formation better since entering the situation could bring the LiFE activity into mind automatically. Next to habits, intrinsic motivation is another beneficial factor for long-term maintenance physical activity behaviour [41]. Self-Determination Theory [38] proposes intrinsic motivation to arise alongside the fulfilment of three psychological needs, autonomy, competence, and connectedness. gLiFE fosters these needs through self-empowering participants to manage their training independently and become their own LiFE trainer. In contrast to LiFE, gLiFE has the potential to foster connectedness particularly through the presence of peers.

The LiFE programme already used the habit formation theory, and while participants' action plans were devised to incorporate elements of habit reforming, it was only taught to trainers not participants. However, increasing participants’ awareness on the psychological factors which can promote behaviour change may increase intervention success. Therefore, the Behaviour Change Technique (BCT) Taxonomy v1 [42] was applied to map the used theories into intervention practice and short theoretical units of 10–15 min length (Fig. 2). This step is essential for large-scale implementation because it enables facilitators without special training in psychology to teach complex theoretical concepts.

The BCTs drawn from Fleig et al. [20] were revised and adapted to the gLiFE concept and the novel contents were categorised by two of the authors (SL, LF). Next to the LiFE-inherent BCTs (e.g., demonstration of the behaviour, BCT 6.1.; behavioural practice/rehearsal, BCT 8.1.), social reward (BCT 10.4.) was added to promote habit formation through positive reinforcement in the group setting. Information about health consequences (BCT 5.1.) was added to foster positive outcome expectancies. A detailed form of delivery [43] of all applied BCTs and their link to the used theories is described in Table 5 in Appendix 1.

#### Pillar III: instruction

A transparent description of how to teach LiFE in a group setting aims to facilitate dissemination of gLiFE and ensures intervention fidelity. Determining methodological standards is essential to streamline content and delivery of gLiFE.

The gLiFE framework draws on experiences and theories from previous studies [19, 20, 23, 33] refined and upgraded for our purposes. Teaching methods aimed to deliver the two main pillars of gLiFE as effective as possible in a group setting. For the purpose of large-scale implementation, gLiFE is designed for any room equipped with chairs. Instruction includes the following subcategories: methods, organisational setting, and applied materials (Fig. 1).

##### Methods

gLiFE was conceptualised for groups of up to 12 participants following recommendations on group size [31] and previous group-based LiFE pilot studies [23]. Based on group simulations and findings from Li and colleagues [23], we considered two trainers—one main and one co-trainer—as necessary for effective delivery and safety. The main trainer explains and demonstrates the theoretical and practical content, leads group discussions, and acts as the main contact person for participants. The co-trainer demonstrates and corrects the activities, documents, helps to shape in discussions, and ensures safety and support, particularly for functionally impaired participants.

Each gLiFE session follows a predefined order which is listed in Fig. 2.

To teach the LiFE activities and the behaviour change theory in the group setting, (motor) learning principles such as *structuring and progression* [44] (BCT 8.7. graded tasks), *repetition and variation* [44] (BCT 8.1. behavioural practice/rehearsal), and *clarity* [45] (BCT 4.1. instruction on how to perform the behaviour) are applied. *Structuring and progression* is based on established learning guidelines and methods such as *from easy to difficult* [29, 30]. For example, stepping over objects is first taught with a flat piece of paper on the floor in order to prevent slips or trips, later on with a cardboard box in order to simulate a real obstacle. The principle *repetition and variation* includes a repetition of previous LiFE activities.

Based on motor learning theory, trainers use a deductive approach for introducing the LiFE activities [29, 46], i.e., predefined and detailed instructions to ensure a correct movement execution of the LiFE activities (BCT 2.2. feedback on behaviour). Several teaching techniques including frontal teaching (BCT 9.1. credible source), group discussions, open questions, and group work [47] are employed in order to teach gLiFE effectively (Table 5 in Appendix 1).

The second pillar is taught using specific methods such as a flipchart to collect participants’ suggestions for daily situations to implement the LiFE activities. Through the presence of peers, participants get a larger repertoire of potential daily situations and are able to support each other in programme implementation. To compensate for the missing home visits, participants visualise themselves performing specific LiFE activities in their home environment (BCT 15.2. mental rehearsal of successful performance). Visualisation as a mental technique [48] has been applied in LiFE [33] and was successfully used in previous physical activity interventions [49, 50] and has been positively evaluated in a meta-analysis [51].

##### Organisational setting

State-of-the art organisational forms for group teaching and group exercising [28] were chosen to facilitate communication of group members and trainers while ensuring safety during exercising (Table 6 in Appendix 2). This includes a circle of chairs with all participants and trainers facing each other. Chairs allow hold and support if needed. Specific organisational settings are used for specific activities (Table 6 in Appendix 2). For instance, for teaching the activity “walking on toes”, participants walk along a wall. This type of practice can easily be transferred to the home environment, e.g., walking in a hallway, with a high level of safety.

##### Materials

The original materials of LiFE such as the LiFE assessment tool, activity planner, and activity counter [33] served as a basis for the design of the gLiFE materials (Table 1). Participants receive a workbook containing a modified activity planner which simplifies the planning and self-monitoring procedure (BCT 2.3. self-monitoring of behaviour). It combines the activity planner with the activity counter because the paperwork has been reported to be tedious in former studies [18, 20, 23]. Since the LiFE activities are identical in LiFE and gLiFE, participants receive the German version of the LiFE participant's manual [52].

In addition, specific materials for teaching LiFE in a group were developed such as a poster with the LiFE principles, posters displaying the different LiFE activities as well as different aids for practicing the correct movement execution (e.g., a poster with a kitchen shelf which we attached to the wall to practice standing on toes). The ideas from the group discussions are collected on flipchart.

## Methods

### Part II: feasibility testing

A feasibility study (ClinicalTrials.gov, NCT03412123) was conducted to test the proof-of-concept of gLiFE. This included an evaluation of the three pillars of the gLiFE concept, i.e., the LiFE activities (pillar I), a pre-post assessment of psychological components related to behaviour change (pillar II) and gLiFE’s instruction (pillar III).

#### Design and setting

A single-group feasibility study was conducted from January to March 2018, including seven weekly gLiFE sessions. A multimodal pre-post assessment approach including quantitative and qualitative feasibility measures as well as exploratory self-reported psychosocial measures was applied.

#### Population

A sample of eight community-dwellers aged 65 years and older was envisaged. They were recruited from a list of former participants of studies conducted at the Network Aging Research in the field of ageing and physical activity. To avoid interferences with our study, we chose participants whose former study participations were at least more than 6 months ago. Eligible participants had to be able to reach the study centre independently and willing to sign written informed consent. Those with an unstable or terminal medical condition, cognitive impairment according to the CogTel questionnaire [53], or severe visual or hearing impairment were excluded.

#### Procedure

Baseline characteristics and outcome measures were assessed prior to group participation at the Network Aging Research (Heidelberg University, Germany). One week before the first group session, participants received the LiFE participant's manual [52]. The gLiFE sessions were delivered in accordance with the developed gLiFE concept (Fig. 2) by an exercise scientist as main trainer (FK) and a psychologist (SL) as co-trainer. The duration of gLiFE sessions ranged from 1.5 to 2 hours. After the intervention phase, outcome measures were obtained.

#### Descriptive measures

Participant characteristics including sex, age, BMI, educational level (highest degree of education), physical activity status (below or above 150 min of moderate to vigorous activity per week in the past 12 months [54]), pain level in the past 4 weeks (6-point Likert scale, no pain to very high pain), impact of pain on activities of daily living, fall history in the past 12 months, fall injuries, perceived fall risk (below average to above average), number of comorbidities, and functional strength (5-chair-rise test [55]) and balance (8-level balance scale [16]) were assessed at baseline.

#### Outcome measures

The outcome measures included quantitative and qualitative feasibility measures.

#### Quantitative feasibility measures

The following quantitative outcomes were assessed using a questionnaire previously developed for evaluating LiFE [19].

##### Perceived safety and adverse events

Participants rated their perceived feeling of safety during the execution of LiFE activities on a 7-point Likert scale. Participants documented adverse events including pain, falls, and injuries during the intervention phase.

##### Adherence

We assessed the average number of participants per session. Based on other LiFE studies [19], participants reported the number of their implemented LiFE activities and weekly frequency of practice as an additional measure of adherence.

##### Acceptability

Participants rated the overall acceptability of gLiFE from 1 (very good) to 6 (insufficient); one question on whether the participants would recommend gLiFE to a friend (yes/no); participants rated (a) the perceived helpfulness of LiFE activities for improving balance, strength, and physical activity; (b) the perceived difficulty of LiFE activities and of upgrading; and (c) the implementability into daily life on a 7-point Likert scale from 1 (not at all) to 7 (very much).

#### Qualitative feasibility measures

A semi-structured focus group interview was conducted to gather further information about structure and content of gLiFE, competence of trainers, used materials, implementation of the LiFE activities and ideas for improving gLiFE. The focus group was administered by an independent researcher not involved in the intervention.

#### Exploratory self-reported psychosocial measures on behaviour change

To get an initial indication on the psychological processes related to behaviour change in gLiFE, selected variables were assessed prior to and post intervention. Response formats of the applied questionnaires were 6-point Likert scales ranging from 1 (completely disagree) to 6 (totally agree), unless stated differently.

*Intention* to practice the LiFE activities and to realise an active lifestyle was assessed using two items (Table 2).

**Table 2:** Exploratory self-reported measures on behaviour change (*N* = 6)

Construct (number of items)	Items (example)	T1 median (IQR)	T2 median (IQR)
Intention (2)	I intend to live an active lifestyle.	6.0 (0.1)	6.0 (0.3)
Self-determined exercise motivation (24)	I exercise because it’s fun.	3.5 (1.4)	4.0 (0.6)
Action and coping planning (4)	During the last week, I have made a detailed plan regarding the situations in which to perform the LiFE activities.	4.5 (1.9)	5.0 (1.4)
Action control (2)	During the last week, I watched carefully to perform the LiFE activities as I planned to.	4.3 (1.4)	3.0 (0.5)
Habit strength (4)	The LiFE activities are something I do automatically.	3.4 (1.3)	4.5 (2.0)

Response format: Intention, action and coping planning, action control and habit strength were assessed on a 6-point Likert scale (1 “completely disagree” to 6 “totally agree”) and self-determined exercise motivation was assessed on a 5-point Likert scale (0 “does not apply to me at all” to 4 “totally applies to me”). T1 was assessed before gLiFE intervention, T2 was assessed post intervention

*Self*-*determined exercise motivation* was assessed using the Behavioural Regulation in Exercise Questionnaire (BRE-Q3 [56]). The questionnaire consists of 24 items measuring six different motivational qualities with four items ranging from 0 (does not apply to me at all) to 4 (totally applies to me). The Relative Autonomy Index (RAI) is a weighted score indicating the level of self-determined motivation. Higher scores indicate higher self-determined motivation.

*Action and coping planning* was assessed using four items according to Sniehotta et al. [57] which were adapted to study purposes.

*Action control* was assessed using two items according to Sniehotta et al. [57].

*Habit strength* was assessed using four items of the Self-Report Behavioural Automaticity Index [58]. The four items were adapted to the LiFE activities, e.g., “*The LiFE activities are something I do automatically*”.

#### Data analysis

Participant characteristics are reported as number of participants (*N*), percentage (%), median, and interquartile range (IQR), as appropriate. Number of implemented LiFE activities and frequency of practice are also reported as median and IQR. Likert scale questionnaires are reported as median and IQR. We used SPSS 24.0 (IBM, Armonk, NY, USA) to calculate descriptive results. Focus group recordings were transcribed and subsequently analysed using an inductive qualitative content analysis [59]. Two authors (FK, SL) independently familiarised themselves with the interviews and built three categories in three subsequent steps. The authors agreed on a set of codes and applied them to the whole manuscript. Subsequently, both authors created a coding network using NVivo11 (QRS International, Australia).

## Results

**Fig. 3:**
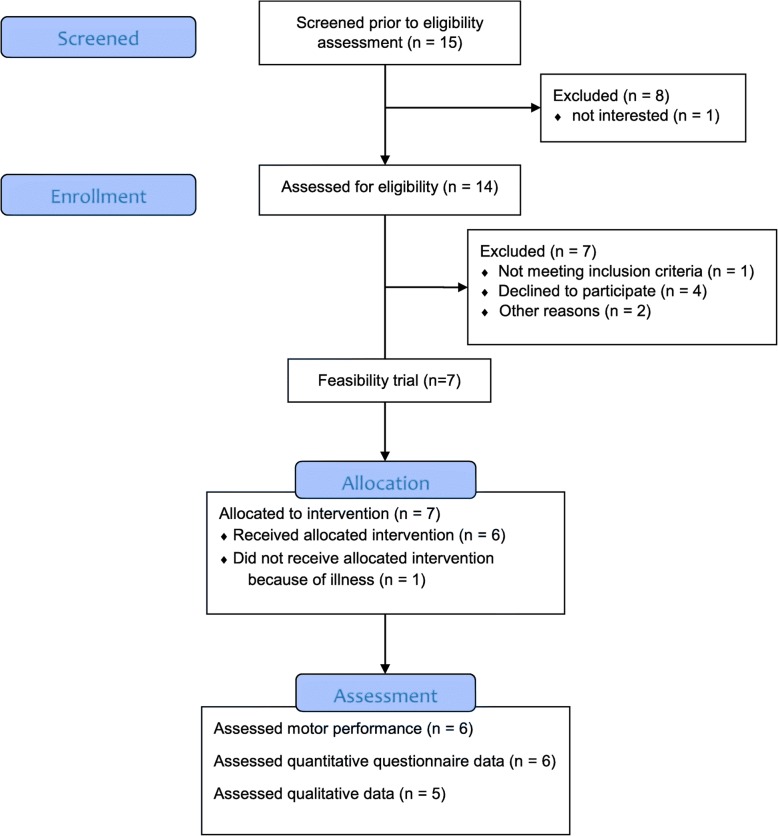
Flow diagram according to the CONSORT guidelines

Seven participants were willing to take part in the study; one participant withdrew due to health problems, six participants (median = 72.8, IQR = 2.8, 5 female) completed the intervention (for the flow diagram, see Fig. 3 in Appendix 3). The sample was heterogeneous with respect to education level, physical activity level, perceived pain, fall history, and comorbidities (Table 3). Participants reported to perceive their risk of falling as being average compared to other persons their sex and age. However, according to the cut-off values for functional strength measured by the 5-chair-rise test [60], our sample had a high risk of falling. Participants’ balance measured by the 8-level balance scale is comparable to previous studies [16]. Participants did not report any major acute health conditions.

**Table 3:** Descriptive characteristics of the study population (*N* = 6)

	*N*	%	Median (IQR)
Sex			
Female	5	83.3	
Male	1	16.7	
Age			72.8 (2.8)
BMI			28.0 (2.3)
Highest degree of education			
Secondary school	3	50.0	
University of applied science	2	33.3	
University degree	1	16.7	
Physical activity (times per week)			
None	2	33.3	
1	3	50.0	
> 1	1	16.7	
Pain level (past 4 weeks)			3.0 (2.0)
Impact of pain on ADLs			3.0 (1.5)
Falls (last 12 months)			
None	3	50.0	
1	1	16.7	
2	1	16.7	
> 2	1	16.7	
Fall injury (last 12 months)			
Yes	1	16.7	
No	5	83.3	
Perceived fall risk			2.5 (1.0)
Comorbidities (number)			2 (1.5)
Functional status			
5 CRT			12.4 (4.2)
8 LBS			5.0 (0.8)

Physical activity level is defined as times of physical activity of moderate to vigorous intensity per week. Pain level is defined as 0 (no pain) to 5 (very high pain). Impact of pain on activities of daily living (ADL) is defined as 1 (never) to 5 (very). Perceived fall risk was defined as 1 (much below average) to 5 (much above average). 5 CRT = 5-chair-rise test; 8 LBS = 8-level balance scale

### Implementation of the gLiFE intervention

gLiFE was delivered as planned including structure of each group session (part I, Fig. 2). Trainers perceived the lower trainer-participant-ratio as feasible and safe. Applied teaching techniques and organisational settings could be carried out as intended (part I, Table 5 in Appendix 1 and Table 6 in Appendix 2). Switching organisational forms was uncomplicated. However, finding individual training levels for all participants using the LiFE assessment tool in group setting was challenging because trainers had to rate and supervise all participants simultaneously. Documentation of action plans (implementation intentions) with the help of the modified activity planner worked well in the group setting. Trainers perceived the designed low-cost material such as card boxes for stepping over objects as helpful and safe.

### Quantitative feasibility measures

The majority of participants reported they felt “very safe” while performing the LiFE activities (Table 4). No adverse events were reported. On average, five out of six participants attended each session. Most of the participants reported that they had implemented more than half of the LiFE activities over the course of the programme for 5 days per week (Table 4). Overall acceptability of gLiFE was “very good” and everyone would have recommended it to a friend. Participants rated gLiFE as (a) “helpful” for improving balance, strength, and physical activity; (b) “low” with respect to perceived difficulty and upgrading of the LiFE activities; and (c) “rather easy” to implement into daily life (Table 4).

**Table 4:** Quantitative results of the feasibility study (*N* = 6)

gLiFE component	Item	Median (IQR)	Range
Safety	Did you feel safe in the group while doing the LiFE activities?	7.0 (0.3)	1 (very unsafe) to 7 (very safe)
Adherence			
Implemented activities (#)		9.5 (4.0)	0 (none) to 14 (all)
Freq. of perf. (days/week)		5.2 (2.1)	0 (never) to 7 (daily)
Acceptability			
Overall grade	Overall, what grade would you give gLiFE?	1.0 (1.0)	1 (very good) to 6 (insufficient)
Helpfulness to increase:	Do you feel that the activities are useful to improve your balance, strength or physical activity?		1 (very useless) to 7 (very useful)
Balance		6.5 (1.0)	
Strength		6.5 (1.0)	
Physical activity		6.0 (0.8)	
Difficulty of upgrading	How easy or difficult was it for you to adapt the LiFE activities to your own training progress?	5.5 (1.3)	1 (very difficult) to 7 (very easy)
Integration into daily life	How easy or difficult was it for you to integrate the LiFE activities into your daily life?	5.5 (2.3)	1 (very difficult) to 7 (very easy)

*Freq*. *of perf*. Frequency of performance

### Qualitative feasibility measures

Five of six participants took part in the semi-structured interview; one participant cancelled due to illness. Qualitative content analysis resulted in three categories: *Format*, *Implementation of Activities*, and *Perceived Intervention Effects*. Format refers to participants’ opinions on the group setting, safety, trainers, materials, and LiFE activities as well as their delivery; implementation of activities refers to habit formation and cues/prompts to which the LiFE activities can be linked; perceived intervention effects refers to physiological and psychosocial changes related to LiFE.

#### Format

##### Group setting

Participants reported that the atmosphere within the group was “very good” (female, aged 73). Participants “felt very comfortable, also with the trainers” (female, aged 70). One participant would have preferred a larger group size. One participant “found it nice to get to know the activities in the group setting. It showed that being a ‘lone warrior’ is not as effective and motivating as being in a group” (female, aged 68).

##### Safety

In line with the quantitative results, participants reported they felt very safe during the gLiFE sessions. The trainer-participant-ratio was perceived as “good” (female, aged 68) and participants reported they felt safe “having both trainers on [their] side” (female, aged 73).

##### Delivery of gLiFE content

It was stated that the gLiFE sessions had a “good and systematic structure” (female, aged 73) and that the structure was “enjoyable and thoughtful” (female, aged 73). One participant remarked that “the balance between theory and practice was suitable and appropriate for [their] age” (female, aged 78). Participants found the repetition of LiFE activities in the beginning of each session was necessary and useful (“I found the repetitions very nice and I recognised whether I had done the exercises correctly or not”, female, aged 70) and that visualisation were a helpful strategy for embedding the LiFE activities into daily routines. Participants emphasised that the movement corrections were “supportive” (female, aged 73), and “important” (female, aged 73); one-to-one corrections were appreciated (“It has been implicitly corrected without anyone being exposed to the group”, female, aged 78).

##### LiFE activities

Participants reported they felt highly autonomous in choosing their LiFE activities (“I can choose those activities for myself which are effective for me and I can benefit from them, because I have a high risk of falling”, female, aged 78).

##### Material

Participants valued the manual as an additional aid next to the explanations during sessions (“If I didn’t know exactly how to execute one LiFE activity, I used the manual and looked it up”, female, aged 68). In contrast, participants reported that working with the activity planner was too complex and unhandy (“I only used it at the beginning, that was too cumbersome and complex for me”, female, aged 70).

#### Implementation of LiFE activities

##### Habit formation

Participants valued action planning as a central and helpful element for the implementation of the LiFE activities into daily life (“Planning in which daily situation I execute the LiFE activities helped me to carrying out the activities [ … ]. They remind me of doing the exercises in these situations”, female, aged 70). Participants described daily routines in which they could “implement the one or the other LiFE activity the whole day” (female, aged 73). Participants remarked that some new movement habits arose during intervention phase (“I do certain LiFE activities every morning and evening in the bathroom”, female, aged 70).

##### Cues/prompts

Participants described that situational cues were helpful to remember performing the LiFE activities (“That makes a lot of sense and it is good for reminding, it caused a wow-effect”, female, aged 68). However, some participants remarked that they did not always perform the LiFE activities in the situation they chose during gLiFE sessions (“I did not do it in specific situations. Sometimes I just did it when it occurred to me”, female, aged 70).

#### Perceived intervention effects

##### Physiological effects

Some participants described reduced pain related to gLiFE participation. In contrast, one participant “[ …] felt pain while standing up from a seated position and walking on heels” (female, aged 70). One participant at high risk of falling remarked that she felt “much safer while walking on the street. [She] did not fall since Christmas, [She was] really proud of [her]self” (female, aged 78).

##### Psychosocial effects

Participants stated that taking part in gLiFE evoked a feeling of fitness, vitality, and a general sense of well-being: “The LiFE activities [were] very helpful. I really feel a sense of well-being in my body. I feel more relaxed, relieved and less or no more pain” (female, aged 73). All participants planned to continue LiFE because of its relevance and necessity (“It would be stupid not to continue with the LiFE activities. I would only harm myself”, female, aged 78).

#### Exploratory self-reported measures on behaviour change

Descriptive results showed that intention to follow an active lifestyle stayed high, whereas self-determined motivation measured by the RAI increased during intervention phase, which may suggest higher levels of intrinsic motivation after gLiFE than before (Table 2). Action and coping planning and habit strength slightly increased over the course of the intervention whereas action control decreased.

## Discussion

We successfully achieved our study aims in terms of developing (part I) and initially testing (part II) a new gLiFE concept designed for large-scale implementation. To the best of our knowledge, this is the first gLiFE concept specifically tailored towards the purpose of a resource-saving dissemination within public health approaches.

### Part I: Development of the gLiFE concept

Building on previous group-based LiFE concepts, our proposed gLiFE concept has several novel features with respect to large-scale implementation including lower trainer-participant-ratio, flexible implementability into different settings, low-cost materials, and a manualised concept designed to standardise structure of content and teaching procedure. We used the established MRC guidelines for developing the gLiFE concept. There are also other frameworks, such as FRAME [61] in order to refine interventions which could be used in further studies.

#### Development process

The MRC guidelines recommend an iterative approach including multiple improvement cycles when developing complex interventions. Having involved users at an early stage of the gLiFE development process allowed us to test initial ideas on the organisational setting and teaching process. Experiences from the user involvement formed the basis for the further development of gLiFE and were discussed in the interdisciplinary team.

One home visit in addition to the group sessions was discussed as an added value to foster efficient implementation of the LiFE activities into daily life but then dismissed due to the required additional costs and resources.

Another feature of gLiFE in favour of cost-effectiveness is the decreased trainer-participant-ratio. Other studies provide evidence for the feasibility of two trainers for a group size of up to 12 older adults [23]. However, less trainer support also poses a potential lack of safety for fall-prone older adults. Therefore, special focus was given to standardised safety guidelines for gLiFE practice. Using chairs and room walls for an additional base of support proved feasible in our study; special and costly equipment such as parallel bars turned out unnecessary. Two trainers were sufficient for delivering gLiFE safely.

Next to reducing costs, we aimed to boost gLiFE’s effectiveness. The application of established theories on group learning helped to compensate for the fact that a simple blueprint of the LiFE is not feasible for the group setting. However, the group setting offers several opportunities such as role modelling [27] or social support which are not present in a one-to-one scenario. In gLiFE, we explicitly made use of group dynamics through implementing group discussions and partner exercises in order to foster the learning process.

Moreover, we used special materials and teaching techniques to ensure the transferability of the LiFE activities from the group setting into participants’ daily lives. For instance, a poster displaying a kitchen shelf allowed to practice LiFE with relation to common furniture and the respective daily situation (e.g., pick something from a shelf). We know that these features seem quite simple and may not solely facilitate successful transfer, which is why we intensified the already existing teaching techniques such as visualisation and intensively discussed possible daily situations with the group.

In summary, during the development process, we made various trade-offs to ensure gLiFE’s cost-effectiveness. The interdisciplinary discourse resulted in a gLiFE concept which contains the core elements of LiFE while having the potential for large-scale implementation. Whether this resource-saving format proves to be similarly effective but less costly compared to LiFE is currently evaluated in a large trial [17].

#### Conceptual gLiFE framework

We optimised the theoretical framework in terms of structure and content in order to increase gLiFE’s long-term effectiveness on basis of current scientific evidence. The first pillar *LiFE Activities and Principles* was maintained, whereas the concept of how to introduce the LiFE activities has been revised. Introducing the LiFE activities gradually and repeating the LiFE activities in the subsequent session allows participants to familiarise themselves with the LiFE activities and test them in daily situations between sessions.

The second pillar *Theory of Behaviour Change and Behaviour Change Techniques* shall ensure the sustainable implementation of LiFE. Theories such as the HAPA or the Self-Determination Theory were not only used to design the theoretical units, but also provided a basis for the teaching aim. For example, participants’ competence was fostered through teaching the LiFE principles and emphasising the reasoning behind the importance of situational cues to the participants in order to create new movement habits. Using implementation intentions to link the daily situation to one specific LiFE activity, as in the original LiFE programme, seems to be a promising tool to boost habit formation.

In contrast to former studies [20] which did not provide specific information on instructing a group-based LiFE format, our manualised gLiFE concept ensures standardised dissemination in a variety of public health settings and improves replicability in scientific studies. Furthermore, gLiFE entails a comprehensive description of the contents on behaviour change and the BCTs and thereby allows their standardised application. Providing trainers with limited psychological background with prepared information on long-term behaviour change might increase gLiFE’s success.

In summary, the new conceptual gLiFE framework not only offers a profound theoretical basis which can be tested in scientific settings, but also provides detailed information on instructing gLiFE which may help to implement gLiFE on a large-scale.

### Part II: Feasibility testing

The gLiFE feasibility testing was carried out as planned. Qualitative and quantitative outcomes obtained via multimodal evaluation suggest that gLiFE is feasible and well-accepted in the target group. Findings are in line with previous studies in young female seniors (mean age 66 years) [20]. We demonstrated that the gLiFE concept is also feasible and accepted in an older sample including individuals at risk of falling and functional impairment who display the key target group of LiFE. Our gLiFE concept could be a resource-saving alternative to LiFE feasible for large-scale implementation.

#### Quantitative feasibility measures

We used established quantitative measures in order to judge the core elements of gLiFE’s feasibility. The fact that gLiFE was generally highly accepted by participants is in line with other LiFE studies [16, 21] and suggests that it is well-suited for the needs and capabilities of the target group. The high attendance rates mirror this finding.

Ensuring safety is one fundamental aspect of feasibility. At the same time, effective balance training requires participants to practice close to their stability limits (overload principle [62]) which has risk-potential in a group of fall-prone older adults. Our developed structure to teach LiFE activities in the group (e.g., two trainers and specific organisational settings) may explain participants’ feelings of safety expressed during the evaluation. The assumption of gLiFE being safe is supported by the fact that no adverse events occurred during group sessions. Likewise, participants did not report any adverse events while practicing the LiFE activities in everyday life, suggesting that participants understood the recommendation for practicing LiFE safely.

The key element of the gLiFE concept are the 14 LiFE activities of which participants may include as many as they like. In the case of lifestyle-integrated training, the number of LiFE activities implemented is both an adherence measure and a marker for behaviour, because the main aim is that participants practice at home independently. The fact that most participants implemented around 75% of LiFE activities is in line with their reported low difficulty of implementing LiFE activities into daily situations. Our finding is comparable to adherence rates from previous studies (76%) implementing LiFE after a one-to-one delivery [19] and measuring adherence in the same manner. This implies that gLiFE facilitates the transfer of LiFE activities from the group setting into daily life. Likewise, the frequency of practicing LiFE—which is highly dependent on the daily situations the activity is liked to—is comparable to previous LiFE studies [12]. This suggests that brainstorming daily situations together via group discussions might be as useful as doing it one-to-one.

In summary, quantitative data suggests that the developed gLiFE concept may be as feasible as LiFE. A direct comparison between LiFE and gLiFE in future studies will clarify if one format is more or less effective.

#### Qualitative feasibility measures

Participants’ positive feedback about the group setting and atmosphere in the focus group suggest that a peer group might be beneficial for evoking feelings of comfort, joy, and motivation [63–65]. The perceived high safety level during sessions is in line with previous studies [23].

The positive feedback about structure, content, and distribution suggests that the gLiFE concept is suitable for the target group. The reported high degree of autonomy when choosing and implementing individual LiFE activities suggests that the gLiFE concept empowered participants to manage their LiFE training independently.

The perceived helpfulness of action planning and identification of situational cues indicates that participants understood these two features to be crucial for habit formation and long-term success with LiFE. The fact that some LiFE activities already became habitual after the intervention phase of 7 weeks supports that raising the importance of habit formation in the theoretical basis (pillar II) is a promising approach for promoting long-term behaviour change. However, some participants reported to perform the LiFE activities independent of their chosen specific daily situation. This might hamper habit formation because repeating the action in the same context is considered essential [35, 66, 67].

The decrease in overall pain and the increase in general feelings of fitness and self-efficacy after gLiFE suggest that gLiFE may not only have an impact on functional status but might also be beneficial regarding overall well-being. These findings are in line with previous studies [16, 21, 65], especially the effects of LiFE on psychosocial factors display an interesting topic for further research.

#### Exploratory self-reported measures on behaviour change

The fact that the intention stayed high over the course of the intervention suggests that participants kept their aim of being active until after intervention. However, a large gap between the intention to engage in physical activity and real physical activity behaviour has been observed [68], which is why intention should not be considered the only predictor for physical activity behaviour. Studies found evidence for self-regulatory strategies being capable of bridging this “intention-behaviour gap” in the physical activity domain [69].

The descriptive increase of self-determined motivation may support the assumption that gLiFE fosters autonomy and thereby fosters self-determined motivation [38] which could contribute to long-term maintenance of the LiFE activities.

The descriptive increase of action planning suggests that participants made use of implementation intentions in order to plan when and where they would implement the LiFE activities into their daily routines. Even though no conclusions can be drawn on these descriptive findings, they can be interpreted as an initial indicator for a successful application of implementation intentions. Other studies did not evaluate the use of implementation intentions particularly, but found planning interventions to be highly useful for the LiFE context [16, 20] as well as for the formation of physical activity habits [70, 71].

The descriptive increase of habit strength after 7 weeks of practice suggests that habit formation was successful in this small sample. This finding is in line with another group-based LiFE pilot study [20] and other studies investigating habit formation in the health behaviour context [34].

### Limitations

In line with the MRC guidelines, this initial feasibility study demonstrates the proof-of-concept of the newly developed gLiFE concept. Large-scale implementability and cost-effectiveness could not be evaluated yet, but a large study building on the present one is currently being carried out [17].

A core element for intervention implementation is fidelity [72]. In this pilot study, trainers reported that intervention implementation was successful, but we did not systematically assess fidelity based on a specific methodology, as this would have required additional resources [21, 22], which were not available in the LiFE-is-LiFE project [17]. Fidelity is certainly a key aspect in larger studies evaluating the gLiFE concept.

The small and selected sample hampers a generalisation of findings. Even though a researcher unknown by participants conducted the focus group, a potential report bias cannot be excluded, as our participants were specifically interested in research project participation. Further, social desirability [73] might have biassed participants’ critical feedback on gLiFE. One-to-one interviews could have revealed more specific information on participants’ opinions.

Despite our effort to simplify the activity planner, some participants still found it complex to handle. This may display a general limitation of paper-pencil-based materials related to LiFE. An ICT-based solution could be a promising alternative [18]. Further, the question remains whether the documentation critique is truly related to the paperwork or a general issue related to behaviour change (i.e., action control).

### Future research

After the development (part I) and initial feasibility testing (part II), the next step is evaluating gLiFE‘s cost-effectiveness and large-scale implementability. In the currently running LiFE-is-LiFE trial, we evaluate these aspects including quantitative and qualitative outcomes on participants’ experiences with gLiFE (e.g., group size, organisational setting, and materials), adherence to LiFE post intervention and behaviour change outcomes such as self-determined motivation and habit formation [17].

## Conclusion

This concept paper presents the development (part I) and initial feasibility testing (part II) of a novel gLiFE concept for community-dwelling older adults at risk of falling. According to the MRC framework, these first two steps are crucial for achieving high quality of complex interventions. The greatest innovation of our study is the first standardised version of a group LiFE concept, including a manual on its conduct. gLiFE is based on a theoretical framework and was specifically designed for large-scale implementation. The successfully completed MRC-based development process in combination with the positive results of the feasibility study demonstrates the proof-of-concept of our approach and justifies proceeding to MRC part III (evaluating gLiFE’s effectiveness). If gLiFE proves itself as effective as or nearly as effective as LiFE, a wide-spread dissemination of gLiFE into public health settings can be advised, fostering older adults’ long-term adherence to fall prevention.

## Data Availability

Data of the feasibility study in shape of pseudomised quantitative (data set) and qualitative (texts) are available upon request to interested researchers. Please submit requests to Dr. Michael Schwenk (schwenk@nar.uni-heidelberg.de), Network Aging Research, Heidelberg University, Germany.

## Appendix 1

**Table 5:** Content, form of delivery and BCTs of gLiFE ordered by first appearance in sessions

Session	Content	Method	Organisation	Material	BCT
1	Introduction of gLiFE	Frontal teaching, open questions	Circle of chairs	Laminated card showing the LiFE team	Information about health consequences^a^ (5.1.)
1, 2	Familiarisation/icebreaker	Group work	Open	Name tags	Social support (unspecified) ^c^ (3.1.)
1–7	Theory-based behaviour change units	Frontal teaching, group discussion, group work, open questions	Circle of chairs	Laminated cards showing the topic, poster with LiFE principles, flipchart	Problem solving^a^ (1.2.), instruction on how to perform the behaviour^c^ (4.1.), information about antecedents^b^ (4.2.), prompts/cues^b^ (7.1.), credible source (9.1.), focus on past success^a^ (15.3.)
1–6	LiFE activities	Frontal teaching, demonstration by trainers	Dependent on LiFE activity (see Table 6 in Appendix 2)	Laminated cards	Feedback on behaviour^c^ (2.2.), social support (unspecified) ^c^ (3.1.), information about health consequences^a^ (5.1.), demonstration of the behaviour^c^ (6.1.), credible source (9.1.), social reward^c^ (10.4.)
1–6	Assessment of LiFE activities	Group assessment	Dependent on LiFE activity (see Table 6 in Appendix 2)	LiFE Assessment Tool	Social reward^c^ (10.4.), graded tasks (8.7.)
1–7	Visualisation	Frontal teaching, individual work	Circle of chairs		Mental rehearsal of successful performance^a^ (15.2.)
2–7	Repetition of LiFE activities	Frontal teaching, demonstration in a group, partner work	Dependent on LiFE activity (see Table 6 in Appendix 2)	Additional material (e.g., towels)	Feedback on behaviour^b,c^ (2.2.), behavioural practice/rehearsal^b^ (8.1.)
2–7	Positive experiences, greatest challenge in execution of LiFE and problem solving	Group discussion	Circle of chairs, semi-circle of chairs		Problem solving^a^ (1.2.), review behaviour goal(s) ^a^ (1.5.), social support (unspecified) ^c^ (3.1.), social reward^c^ (10.4.), focus on past success^a^ (15.3.)
1–7	Action planning	Group discussion, individual work	Circle of chairs, semi-circle of chairs	Flipchart, laminated cards showing LiFE activities, flipchart	Goal setting^a^ (behaviour) (1.1.), action planning^a^ (1.4.), prompts/cues^b^ (7.1.)
1–7	Wrap-up/summary	Frontal teaching, open questions, group discussion	Circle of chairs, semi-circle of chairs		

*BCT* Behaviour change technique. Behaviour change theory is linked to the different BCTs as the following: ^a^*HAPA*, ^b^Habit formation theory, ^c^Self-Determination Theory

## Appendix 2

**Table 6:** Organisational forms for teaching the 14 LiFE activities in gLiFE

	Circle of chairs	Semi-circle of chairs	Semi-double-circle of chairs	Row (of chairs)
Session 1				
Tandem stand	X	X		
Tandem walk	X			
Sit to stand		X	X	
Squatting		X	X	
Session 2				
Leaning				X
Standing/walking on toes	X			X
Session 3				
Stepping over objects	X			
Standing/walking on heels	X			X
Session 4				
Climbing stairs				X
Session 5				
One-leg stand	X	X		
Tighten muscles	X	X		
Session 6				
Move sideways	X			

## Abbreviations

BCTs: Behaviour Change Techniques
BMI: Body mass index
BRE-Q3: Behavioural Regulation in Exercise Questionnaire 3
gLiFE: Group-based Lifestyle-integrated Functional Exercise
HAPA: Health action process approach
IQR: Interquartile range
LiFE: Lifestyle-integrated Functional Exercise
MRC: Medical Research Council
RAI: Relative Automaticity Index
